# Tamoxifen Promotes Sustained *Bifidobacterium* Enrichment in the Gut Microbiota of Male and Female Rats After Spinal Cord Injury

**DOI:** 10.3390/ijms27198729

**Published:** 2026-09-29

**Authors:** Luis H. Pagán-Rivera, Filipa Godoy-Vitorino, Samuel E. Ocasio-Rivera, Maria E. Santiago-Gascot, Viviana González, José M. Santiago, Iris Salgado, Aranza Torrado-Tapias, Jennifer M. Colón-Mercado, Carolina García-Guay, Jorge D. Miranda

**Affiliations:** 1 Department of Physiology, University of Puerto Rico Medical Sciences Campus, San Juan, PR 00936, USA; luis.h.pagan.rivera@upr.edu (L.H.P.-R.); samuel.ocasio@upr.edu (S.E.O.-R.); maria.santiago22@upr.edu (M.E.S.-G.); viviana.gonzalez5@upr.edu (V.G.);; 2 Department of Microbiology and Immunology, University of Puerto Rico Medical Sciences Campus, San Juan, PR 00936, USA; 3 Department of Natural Sciences, University of Puerto Rico Carolina Campus, Carolina, PR 00984, USA; jose.santiago13@upr.edu; 4 School of Medicine, Universidad Central del Caribe, Bayamon, PR 00956, USA; iris.salgado@uccaribe.edu; 5 National Institutes of Health, National Human Genome Research Institute, Bethesda, MD 20892, USA; aranza.torrado-tapias@nih.gov; 6 Department of Psychology, University of Puerto Rico Rio Piedras Campus, San Juan, PR 00926, USA; carolina.garcia5@upr.edu

**Keywords:** microbiome, trauma, selective estrogen receptor modulator, anti-inflammatory, neuroprotective, gut–brain axis

## Abstract

Spinal cord injury (SCI) triggers complex secondary injury cascades that limit neurological recovery. Tamoxifen (TAM), a selective estrogen receptor modulator, exerts neuroprotective effects in experimental SCI, although its underlying mechanisms remain unknown. Given the emerging role of gut microbiota dysregulation in SCI, we investigated whether TAM-associated recovery is accompanied by alterations in gut microbial composition and predicted function. We conducted a longitudinal, sex-inclusive analysis of 61 Sprague–Dawley rats of both sexes assigned to naïve (NAÏVE), sham-placebo (SHAM PLB), sham-TAM (SHAM TAM), injured-placebo (INJ PLB), or injured-TAM (INJ TAM) groups. Injured animals received a moderate T10 contusion followed by a 21-day TAM (0.71 mg/day) or placebo pellet, and fecal samples were collected at 0, 7, 14, 21, and 28 days post-injury. Fecal samples were analyzed by 16S rRNA gene sequencing. Results showed that TAM induced sustained shifts in microbial community composition, with treatment-associated divergence emerging at 14 days post-injury (DPI) in females and 21 DPI in males. *Bifidobacterium* exhibited the most consistent TAM-associated enrichment across sexes. *Bifidobacterium* abundance was negatively associated with recovery in INJ PLB animals and positively associated with INJ TAM animals; because behavioral and microbiome measurements were not made in the same animals, these associations are hypothesis-generating only. PICRUSt2 analysis revealed TAM-associated changes in predicted microbial function, including increased glycogen degradation pathways from 14 DPI onward in both sexes. Together, these findings identify sustained TAM-associated taxonomic and predicted functional remodeling of the gut microbiota following SCI.

## 1. Introduction

Spinal cord injury (SCI) is caused by traumatic damage to the spinal cord and can result in temporary or permanent loss of neurological function, accompanied by autonomic dysregulation [1]. To date, a viable treatment for SCI patients remains unavailable, mainly due to complex molecular events centered at the lesion site, which generate a hostile, non-permissive environment [2]. The dynamic SCI pathophysiology can be divided into three phases. The primary (acute) phase spans approximately from the first 2 h to 2 days after injury [3]. During the acute phase, key pathological events include necrosis, inflammation, edema, and hemorrhage. This phase is characterized by mechanisms such as oxidative stress, ionic imbalance, glutamate excitotoxicity, and vascular damage, all of which contribute to the progression of cellular death [4]. The intermediate phase post-SCI begins approximately three days after the injury and may extend for several days [5]. This stage is marked by the infiltration of macrophages as well as B and T lymphocytes. Antigen-presenting cells activate CD4+ helper T-cells, which release proinflammatory cytokines that stimulate B cell activation and antibody production. This immune response amplifies neuroinflammation and exacerbates tissue damage [6]. The chronic phase that takes place days to months after the injury is characterized by apoptosis, demyelination, and the formation of the glial scar, which serves as both a physical and chemical barrier with inhibitory signals for axonal regeneration. However, the glial scar also serves a protective role by limiting lesion expansion and restricting the spread of inflammatory mediators to adjacent intact tissue [7].

Given the varied nature of these molecular events, there is a need for multi-active agents capable of targeting multiple components of the intermediate and chronic phases. The use of estradiol has been shown to confer neuroprotection after central nervous system (CNS) trauma and disease [8,9,10]. However, the use of estradiol has been linked to potentially harmful side effects, including a higher risk of stroke and breast cancer [11]. Tamoxifen (TAM) is a nonsteroidal, triphenylethylene-derived selective estrogen receptor modulator (SERM) that binds estrogen receptors despite lacking the steroid backbone of estradiol, and that has demonstrated neuroprotective effects in experimental models of SCI [12,13,14]. Additionally, TAM functions as an estrogen receptor agonist within the CNS and uterine tissues, while acting as an antagonist in mammary tissue—a dual action that makes it a promising candidate for treating SCI, offering neuroprotection without the systemic hormonal side effects associated with traditional estrogen therapy [15].

Used for breast cancer treatment, this SERM suppresses the activation of estrogen-responsive genes, including those involved in tumor cell-derived growth and angiogenesis [16]. Beyond its clinical use in oncology, TAM has gained attention in the field of neurotrauma due to its protective effects within the central nervous system [17]. After its administration, TAM is converted to its two primary active metabolites, 4-hydroxytamoxifen and endoxifen (4-hydroxy-N-desmethyl tamoxifen), through metabolism by the cytochrome P450 enzyme CYP2D6 [18]. These metabolites act on estrogen receptors in neural tissue, exhibiting anti-inflammatory and neuroprotective properties [19,20]. TAM metabolites may bind to estrogen receptors (ERs) and act as either agonists or antagonists, depending on the presence of specific co-activators and co-repressors within target cells [21]. TAM may therefore act on CNS cell populations expressing ERα, ERβ or GPER1, although the cell types mediating its effects after SCI have not been established. For instance, in a recent 2024 study, Owoyemi et al. demonstrated the neuroprotective properties of TAM in adult male rats exposed to manganese-induced neurotoxicity. TAM-treated animals showed significantly enhanced exploratory behavior and improved locomotor performance, suggesting a protective effect against neurodegeneration [22]. Additionally, earlier work by Valdovinos et al. (2016) revealed that TAM administration in a male feline model of demyelination accelerated remyelination in vivo [12]. These findings support the concept that promoting CNS myelin regeneration is a critical therapeutic approach for mitigating the long-term impact of demyelinating disorders such as multiple sclerosis and further highlight the beneficial effects of TAM in CNS-related pathological conditions.

Numerous studies from our laboratory and others have demonstrated that TAM administration following SCI promotes axonal preservation, exerts neuroprotective and restorative effects, spares neuronal tissue, reduces gliosis, and reduces inflammation, thereby promoting functional locomotor recovery in both male and female rat models [14,15,23,24,25]. However, in our laboratory, we demonstrated that TAM produces its neuroprotective effects in a sex-specific manner with a defined therapeutic window of up to 24 h after SCI in female rats and only 6 h in male rats [26,27]. These findings underscore a sex-dependent therapeutic window for TAM-mediated neuroprotection after SCI. Despite these promising outcomes, the cellular and molecular mechanisms by which TAM exerts its neuroprotective effects remain unknown, representing a critical gap in the field. Elucidating this mechanism is essential for optimizing TAM’s clinical application and for tailoring sex-specific therapeutic strategies.

One emerging mechanism that may underlie TAM’s neuroprotective effects involves the modulation of the gut–brain axis. The gut–brain axis is a bidirectional communication network linking the gastrointestinal tract and the CNS through neural, immune, endocrine, and metabolic pathways [28]. This complex signaling system enables the gut microbiota to influence brain function, neuroinflammation, and neuroplasticity [29,30]. The gut microbiota itself is a vast and dynamic community of trillions of microorganisms that reside along the gastrointestinal tract. Beyond its local effects on digestion and metabolism, the gut microbiota contributes to host homeostasis through the synthesis of bioactive metabolites, maintenance of epithelial barrier integrity, modulation of systemic immune responses, and communication with the CNS via the gut–brain axis [31,32]. SCI commonly induces a state of dysbiosis, an imbalance in the gut microbial community, characterized by a reduction in beneficial anti-inflammatory taxa and an increase in pro-inflammatory species [33]. This dysbiosis is closely linked to compromised intestinal epithelial barrier integrity, which allows the translocation of microbial products into circulation and exacerbates systemic and neuroinflammation [34]. Increasing evidence supports a critical role for the gut microbiota in shaping neuroinflammatory responses, immune regulation, and functional recovery after SCI [35,36,37,38]. Importantly, the gut microbiota also participates in estrogen metabolism through the estrobolome, which comprises gut microorganisms and their microbial genes involved in the deconjugation and metabolism of estrogens, thereby influencing systemic estrogen bioavailability and signaling [39]. Since TAM is a SERM, its administration could theoretically alter the gut microbial ecosystem through interactions with the estrobolome.

We therefore hypothesize that TAM administration after SCI may alter the gut microbiota, either attenuating or exacerbating SCI-associated dysbiosis, depending on its interaction with estrogen-metabolizing gut bacteria. The present study was designed to determine whether TAM administration following SCI could be associated with alterations in gut microbiota composition over time, and whether these changes are consistent with attenuation or exacerbation of SCI-associated dysbiosis. Using a rat model of moderate spinal contusion injury, we characterized longitudinal microbial changes in both male and female animals to evaluate potential sex-specific responses to TAM treatment.

## 2. Results

### 2.1. Study Design and Sample Collection

**Figure 1:**
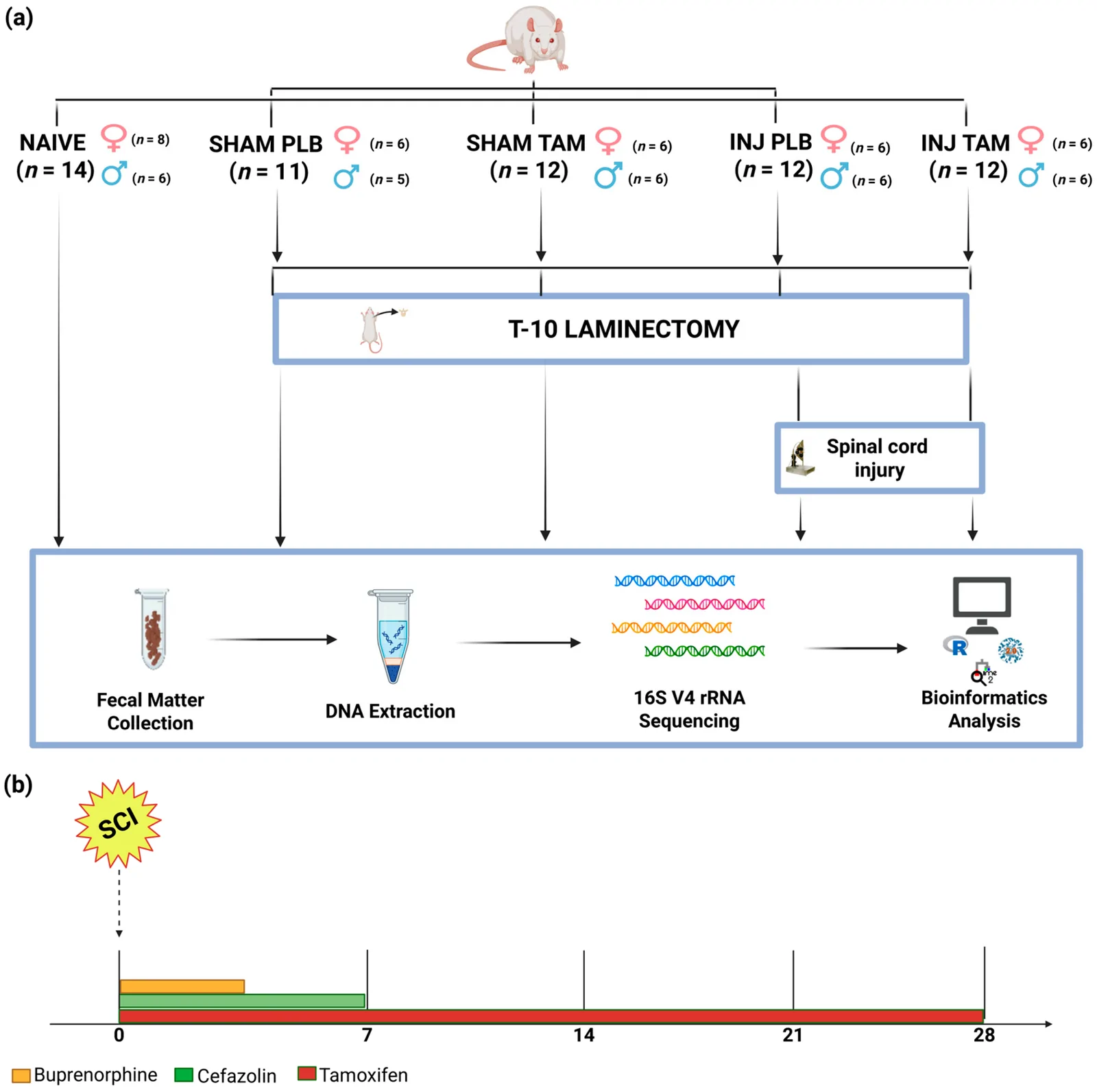
Experimental design and treatment timeline. (**a**) Female and male Sprague–Dawley rats were assigned to five experimental groups: NAÏVE, SHAM_PLB, SHAM_TAM, INJ_PLB, and INJ_TAM. SCI was induced at T10 using the NYU/MASCIS impactor. Fecal samples were collected at days 0, 7, 14, 21, and 28 for 16S rRNA gene sequencing and microbiome analysis. (**b**) Treatment timeline showing TAM pellet administration (0.71 mg/day for 21 days) and post-operative buprenorphine and cefazolin administration. Created with BioRender.com. Pagan, L. (2026). https://BioRender.com/wm3ixmo.

To evaluate the impact of tamoxifen (TAM) administration after spinal cord injury (SCI) on gut microbiota composition, and to determine whether these effects differ between sexes, we conducted 16S rRNA gene sequencing analysis using fecal samples collected from both female and male Sprague–Dawley rats across five timepoints. Animals of both sexes were allocated to five experimental groups: NAÏVE, which received no intervention; SHAM PLB and SHAM TAM, which underwent T10 laminectomy without spinal cord injury (SCI) and received placebo (PLB) or tamoxifen (TAM), respectively; and INJ PLB and INJ TAM, which received a moderate spinal cord contusion following T10 laminectomy and were treated with PLB or TAM, respectively. The planned sample size was six animals of each sex per group. However, 61 animals were ultimately enrolled: 32 females and 29 males. The four surgical groups included 24 females and 23 males, with 5–6 animals of each sex per group, whereas the NAÏVE group included eight females and six males. (Figure 1a). The number of samples retained for individual microbiome analyses varied across groups and timepoints following sequencing quality control and rarefaction to a depth of 4000 reads per sample, as detailed in Table S1. TAM pellets were administered to deliver a sustained release of 0.71 mg/day over a 21-day period. All animals undergoing surgery (SHAM and INJ groups) received cefazolin (25 mg/kg i.p., twice daily for 7 days) and buprenorphine (0.05 mg/kg i.p., twice daily for 3 days); NAÏVE animals received no surgical or pharmacological intervention. Fecal pellets were collected on days 0 (before intervention), 7, 14, 21, and 28 post-injury (Figure 1b).

### 2.2. Beta Diversity Analysis Reveals Tamoxifen-Induced Shifts in Gut Microbiota Composition

To evaluate global differences in the gut microbial community among research groups, we performed beta diversity analysis using Principal Coordinates Analysis (PCoA) based on Bray–Curtis dissimilarity. This analysis was conducted separately for female and male rats across all five experimental groups, incorporating samples from all evaluated timepoints (0, 7, 14, 21, and 28 DPI), and groups effects were assessed using a one-factor PERMANOVA model comparing NAIVE, PLB and TAM groups, with sex analyzed separately.

**Figure 2:**
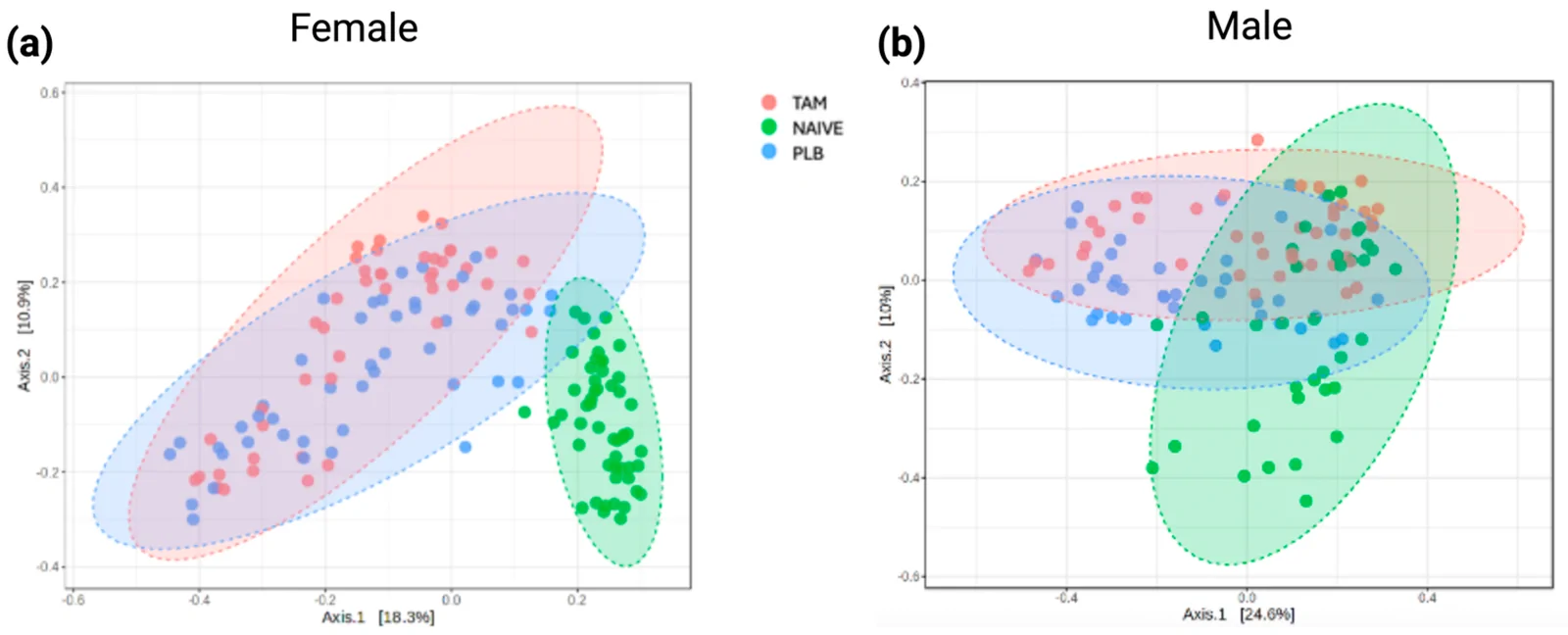
Beta diversity analysis of gut microbiota in female and male rats. Principal Coordinate Analysis (PCoA) based on Bray–Curtis dissimilarity showing fecal microbial community composition in female (**a**) and male (**b**) rats. The analysis includes samples collected across all evaluated timepoints (0–28 days), grouped according to treatment as NAÏVE (green), PLB (blue), and TAM (red). Each point represents an individual fecal sample. Statistical differences in microbial community composition were assessed by PERMANOVA.

In female rats (Figure 2a), distinct clustering patterns were evident among all three groups. The NAÏVE group formed a separate cluster, showing significant divergence from both the PLB and TAM groups (PERMANOVA *p* = 0.001 for both comparisons). Moreover, the PLB and TAM groups also differed significantly from each other (*p* = 0.002), suggesting that TAM alone induces microbial shifts. Group differences in females were supported by PERMANOVA (F = 13.816, R^2^ = 0.161, *p* = 0.001). However, multivariate dispersion also differed significantly among groups (PERMDISP, F = 52.63, *p* < 0.001), indicating that differences in within-group dispersion may contribute to the observed PERMANOVA effect. In males (Figure 2b), distinct clustering was also observed, although separation among groups appeared less pronounced than in females. The NAÏVE group differed significantly from both the PLB and TAM groups (pairwise PERMANOVA, *p* = 0.001 for both comparisons), while PLB and TAM groups also differed significantly from each other (*p* = 0.001); the overall group effect was significant in males (PERMANOVA, F = 6.928, R^2^ = 0.105, *p* = 0.001). In contrast to females, multivariate dispersion did not differ significantly among male groups (PERMDISP, F = 1.499, *p* = 0.227), supporting the interpretation that the male PERMANOVA result primarily reflects differences in community centroid rather than heterogeneity of dispersion. Together, these findings demonstrate significant differences in gut microbial community structure among NAÏVE, PLB, and TAM groups in both sexes, although the significant heterogeneity of dispersion observed in females warrants cautious interpretation of the female PERMANOVA results.

### 2.3. Longitudinal Beta Diversity Volatility Analysis Reveals Increased Microbial Stability in Tamoxifen-Treated Animals

**Figure 3:**
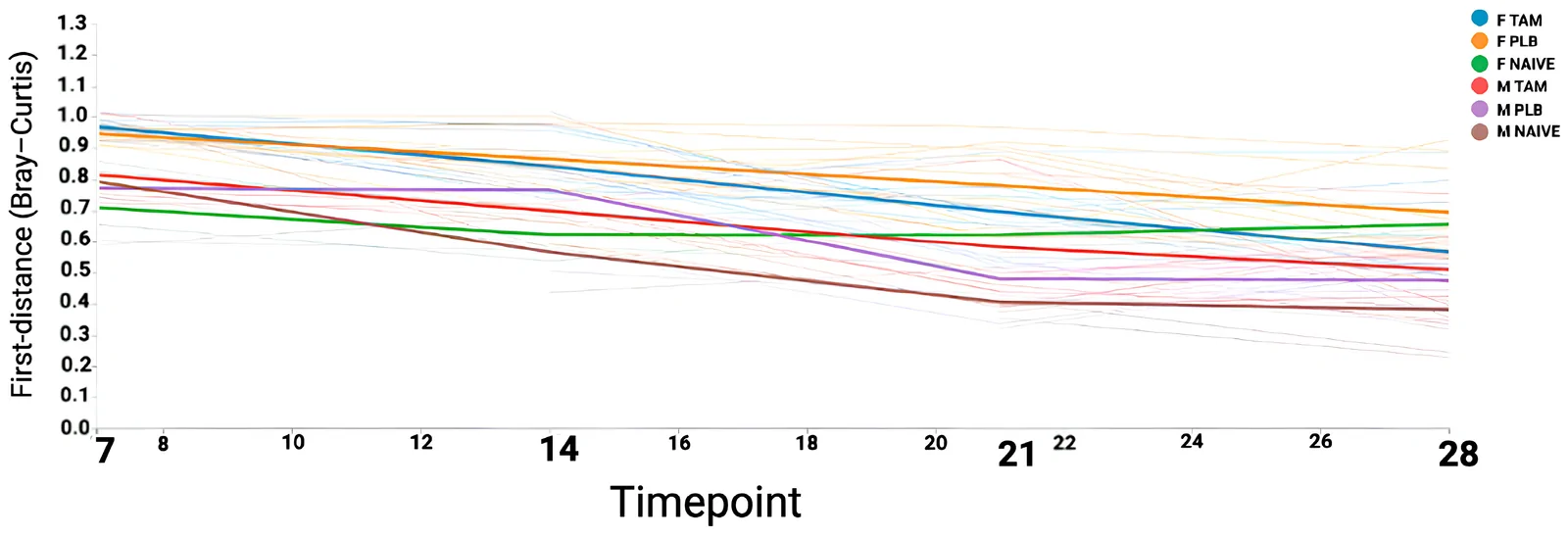
Longitudinal Bray–Curtis dissimilarity between consecutive sampling timepoints following spinal cord injury. Distances were calculated between consecutive sampling timepoints for each animal (day 7 vs. day 0, day 14 vs. day 7, day 21 vs. day 14, and day 28 vs. day 21). Lower Bray–Curtis dissimilarity values indicate greater similarity between consecutive timepoints, whereas higher values indicate greater temporal changes in microbial community composition. Colored lines represent group mean trajectories, and shaded areas indicate the 95% confidence intervals. Blue: female TAM (F TAM); orange: female placebo (F PLB); green: female NAÏVE (F NAÏVE); red: male TAM (M TAM); purple: male placebo (M PLB); brown: male NAÏVE (M NAÏVE).

To evaluate longitudinal changes in gut microbiota composition following SCI, first-distance analysis based on Bray–Curtis dissimilarity was performed using the QIIME 2 q2-longitudinal plugin (Figure 3). Bray–Curtis dissimilarity was calculated between each pair of consecutive samples from the same animal (day 7 vs. 0, 14 vs. 7, 21 vs. 14, 28 vs. 21). From 14 to 28 DPI, female TAM animals exhibited a significant progressive decrease in Bray–Curtis first-distance (β = −0.0193, 95% CI −0.0249 to −0.0138, *p* < 0.001), while female PLB animals showed a comparatively more modest decline (β = −0.0123, 95% CI −0.0193 to −0.0053, *p* < 0.001). In contrast, the female NAÏVE group remained relatively stable over this interval (β = 0.0024, 95% CI −0.0032 to 0.0080, *p* = 0.403). Among males, significant decreases were observed in both TAM (β = −0.0129, 95% CI −0.0241 to −0.0018, *p* = 0.023) and PLB animals (β = −0.0193, 95% CI −0.0275 to −0.0111, *p* < 0.001), as well as in the NAÏVE group (β = −0.0117, 95% CI −0.0214 to −0.0019, *p* = 0.019). Collectively, these findings demonstrate distinct temporal patterns of microbiome dissimilarity across sex and treatment groups, with the most pronounced longitudinal decline among females observed in TAM-treated animals.

### 2.4. Tamoxifen Preserves Firmicutes-to-Bacteroidetes (F/B) Ratio After SCI in Both Sexes

**Figure 4:**
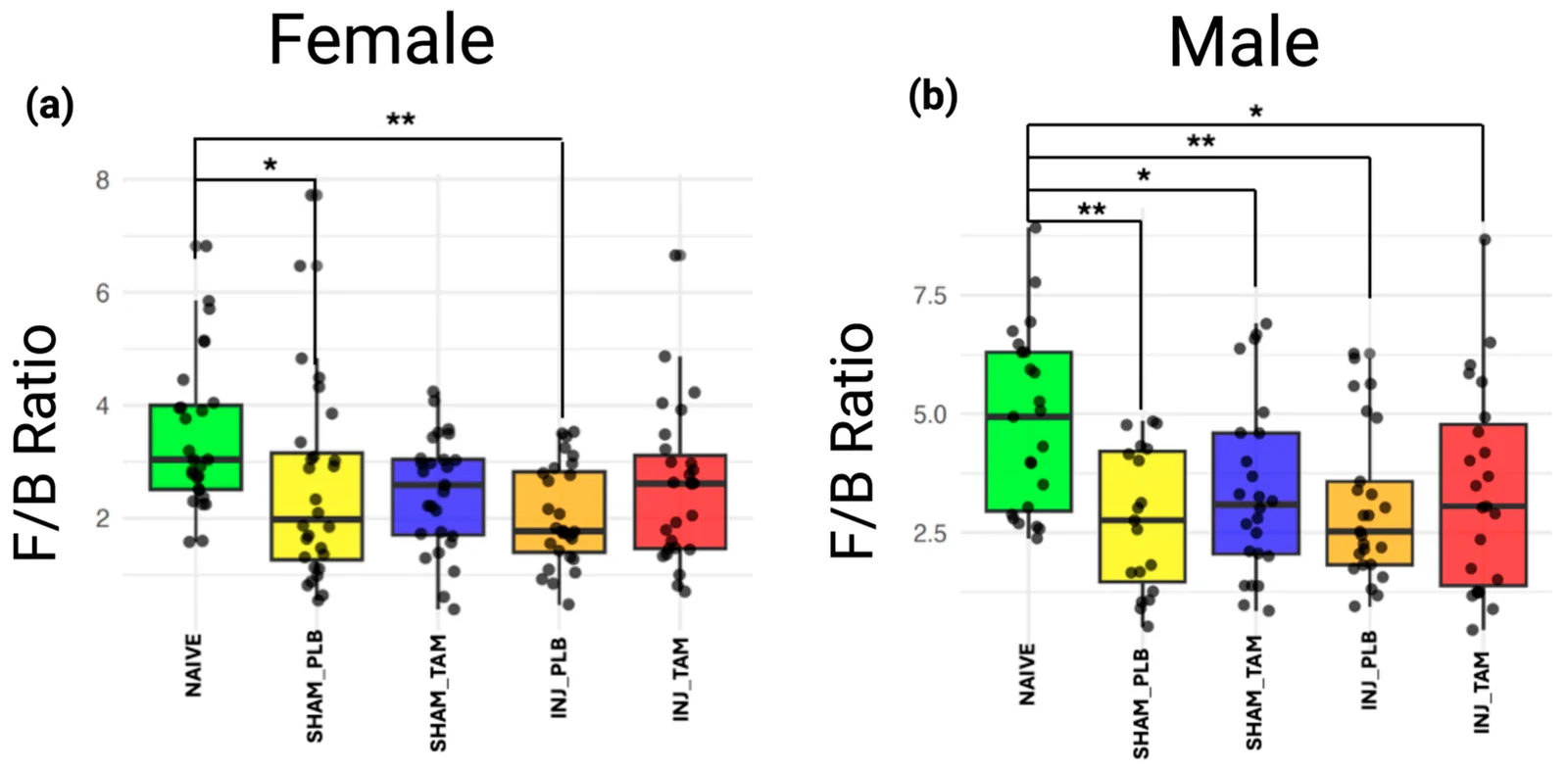
Firmicutes/Bacteroidetes (F/B) ratio across experimental groups following SCI. Boxplots showing the Firmicutes/Bacteroidetes (F/B) ratio in female (**a**) and male (**b**) rats. Experimental groups included NAÏVE, SHAM_PLB, SHAM_TAM, INJ_PLB, and INJ_TAM. Boxplots represent the median and interquartile range, with whiskers indicating the minimum and maximum values. Statistical significance was assessed using the Kruskal–Wallis test followed by Dunn’s multiple comparisons test (* *p* < 0.05, ** *p* < 0.01). Sample sizes (n) indicate the number of fecal samples retained for analysis at each timepoint following sequencing quality control; samples were collected longitudinally from the same animals across the experimental period: NAÏVE (n = 52), SHAM_PLB (n = 24), SHAM_TAM (n = 23), INJ_PLB (n = 23), and INJ_TAM (n = 23); males: NAÏVE (n = 36), SHAM_PLB (n = 19), SHAM_TAM (n = 19), INJ_PLB (n = 22), and INJ_TAM (n = 21).

The Firmicutes-to-Bacteroidetes (F/B) ratio is widely used as an indicator of gut dysbiosis, with alterations often linked to inflammation, although some consider it context-dependent. To further support observed shifts in microbial composition, we evaluated the F/B ratio across the five experimental groups (Figure 4). In females (Figure 4a), both PLB-treated groups (INJ PLB and SHAM PLB) showed a marked reduction in the F/B ratio compared to NAÏVE (** *p* < 0.01 and * *p* < 0.05, respectively), while both TAM-treated groups (INJ TAM and SHAM TAM) maintained values similar to the NAÏVE group. In male rats (Figure 4b), all four surgical groups exhibited significantly lower F/B ratios than the NAÏVE group. The differences were significant at adjusted *p* < 0.01 for the PLB-treated groups (INJ PLB and SHAM PLB) and at adjusted *p* < 0.05 for the TAM-treated groups (INJ TAM and SHAM TAM). However, direct comparisons between the corresponding TAM- and PLB-treated groups were not statistically significant (adjusted *p* > 0.05). Direct comparisons between TAM- and PLB-treated groups were not statistically significant, so the F/B data do not by themselves establish a treatment effect; they are, at most, directionally consistent with the community-level differences described above.

### 2.5. Taxonomic Profile Analyses Reveal Tamoxifen Treatment Modifies Gut Microbial Composition at the Phylum and Genus Levels

**Figure 5:**
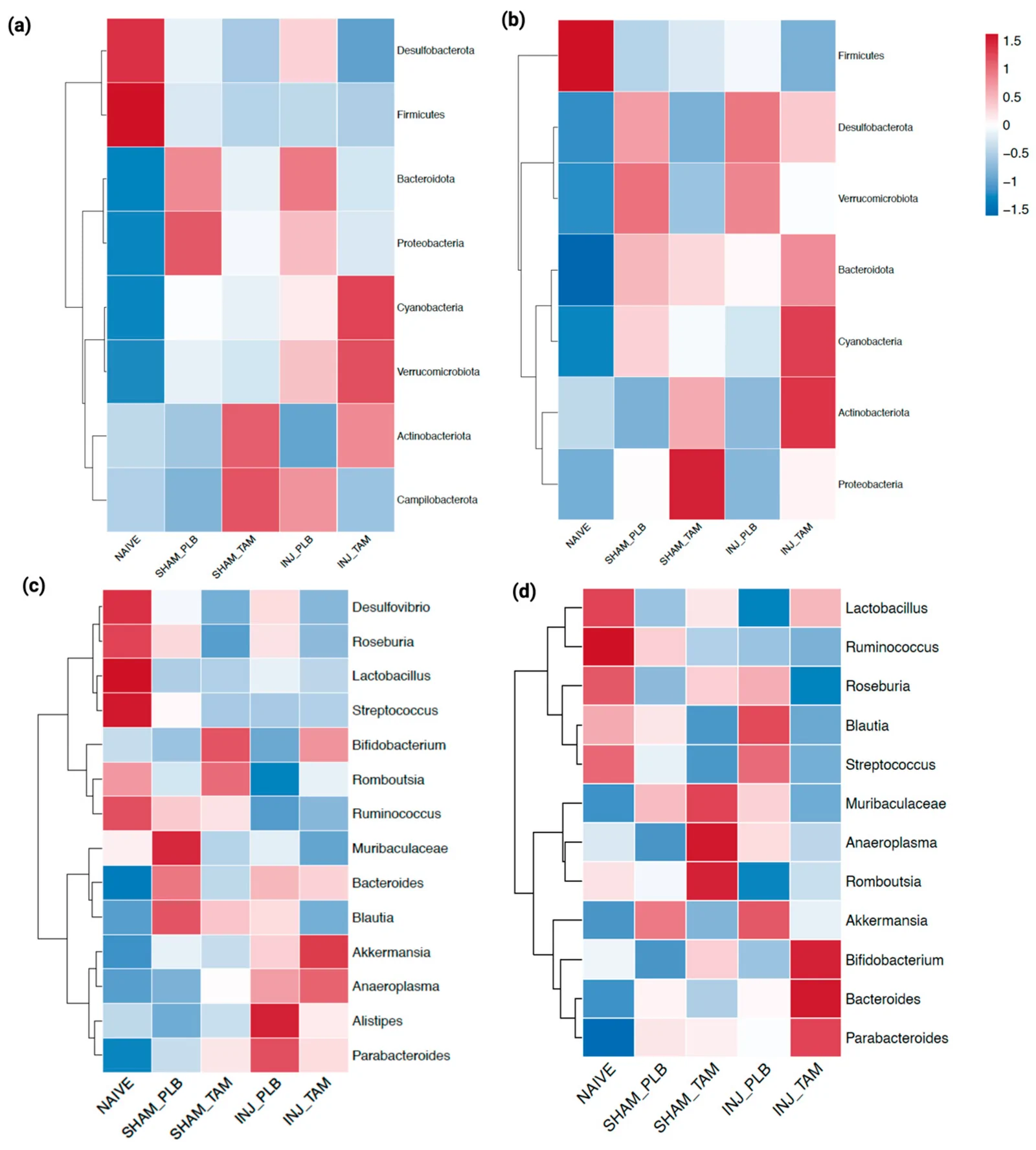
Heatmap of the average relative abundance of bacterial taxa across experimental groups. Heatmaps showing the mean relative abundance of bacterial taxa at the phylum and genus levels in female and male rats. Phylum-level composition is shown for females (**a**) and males (**b**), while genus-level composition is shown for females (**c**) and males (**d**). Cell color represents the row-scaled (z-score) mean relative abundance of each taxon across the five experimental groups, with blue indicating values below and red values above the taxon’s across-group mean. Rows were clustered using Euclidean distance and Ward’s linkage.

For further insight into TAM-induced microbiome alterations after SCI, we performed taxonomic analyses for female (Supplementary Figure S1a,b) and male rats (Supplementary Figure S2a,b). Hierarchical clustering heatmap analysis of relative microbiome abundance at the phylum and genus levels in both sexes was also conducted using Euclidean distance and Ward’s linkage method. At the phylum level, TAM-treated (SHAM TAM and INJ TAM) female rats (Figure 5a) exhibited increased relative abundance of Actinobacteriota (formerly Actinobacteria) compared with PLB and NAÏVE controls. A similar elevation was observed in TAM-treated male rats (Figure 5b), suggesting a conserved response to treatment at the phylum level in both sexes. At the genus level, female TAM-treated groups showed increased abundance of *Bifidobacterium* and *Parabacteroides*, while showing a decrease in *Roseburia* and *Desulfovibrio* (Figure 5c). In males, TAM administration was associated with elevated levels of *Parabacteroides*, *Lactobacillus*, and *Bifidobacterium*, alongside decreased *Blautia* and *Streptococcus* (Figure 5d). These compositional changes further support the microbiome modulatory effects of TAM.

### 2.6. Longitudinal Abundance Patterns of Selected Gut Bacterial Genera

**Figure 6:**
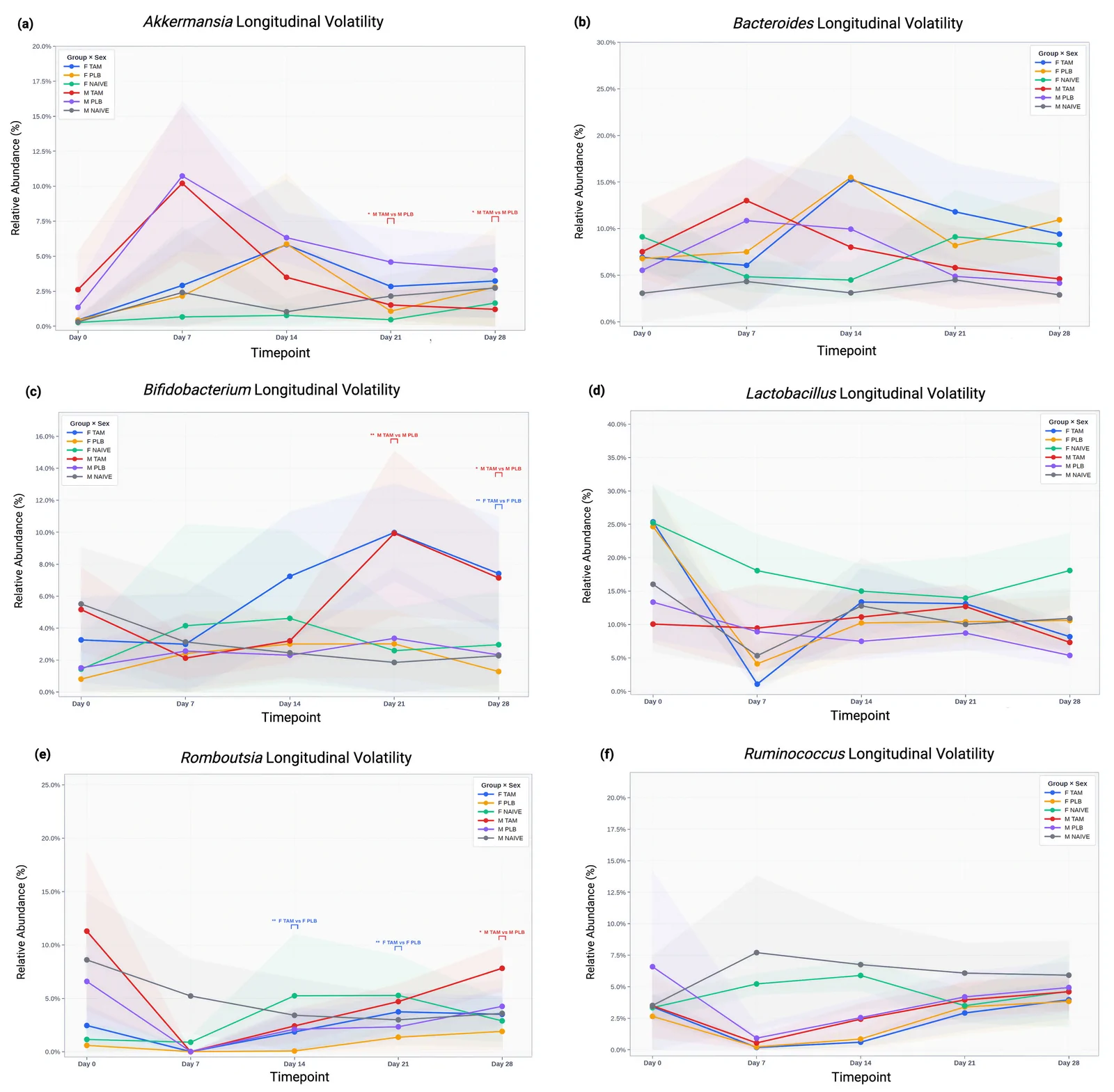
Longitudinal abundance trajectories of selected bacterial genera following TAM treatment after spinal cord injury. Relative abundance (%) of representative bacterial genera was assessed longitudinally in female (F) and male (M) rats from naïve (NAÏVE), placebo-treated (PLB), and tamoxifen-treated (TAM) groups at baseline (Day 0) and 7, 14, 21, and 28 days post-injury (DPI). Panels show (**a**) *Akkermansia*, (**b**) *Bacteroides*, (**c**) *Bifidobacterium*, (**d**) *Lactobacillus*, (**e**) *Romboutsia*, and (**f**) *Ruminococcus*. Solid lines represent the mean relative abundance at each timepoint, and shaded regions indicate the standard deviation (SD). Statistical significance between TAM- and PLB-treated animals within the same sex at individual timepoints was determined using two-sided Mann–Whitney *U* tests performed on individual animal relative abundance values. * *p* < 0.05, ** *p* < 0.01. Group sizes (samples/animals): F TAM 46/12, F PLB 47/12, F NAÏVE 52/8, M TAM 40/12, M PLB 41/11, and M NAÏVE 36/6.

To further characterize temporal microbial dynamics following SCI and TAM administration, longitudinal abundance trajectories of selected bacterial genera with sufficient prevalence and consistent abundance across the study period were evaluated using the volatility analysis implemented in the QIIME 2 longitudinal plugin (Figure 6). While several genera displayed transient injury-associated fluctuations during recovery, only *Bifidobacterium* demonstrated a sustained increase associated with TAM treatment in both female and male rats. *Akkermansia* exhibited a transient increase during the acute post-injury period, particularly in male animals, with abundance peaking at day 7 before progressively declining toward baseline by day 28 (Figure 6a). Although TAM-treated groups showed modest differences compared with PLB animals at later timepoints, these changes were sex-specific males exhibited significantly lower abundance in TAM-treated animals compared with PLB controls at both 21 and 28 DPI, indicating a sustained difference between treatment groups at these later timepoints. Likewise, *Bacteroides* displayed temporal fluctuations following SCI but did not exhibit a consistent treatment-associated pattern in either sex (Figure 6b). In contrast, *Bifidobacterium* displayed a distinct treatment-associated trajectory (Figure 6c). Relative abundance remained low and comparable across all groups through day 7, after which TAM-treated animals exhibited a progressive increase beginning on day 14. Female TAM-treated rats demonstrated an earlier increase, whereas male TAM-treated animals exhibited a delayed but comparable expansion by day 21. Pairwise comparisons confirmed significantly greater *Bifidobacterium* abundance in TAM-treated animals compared with PLB controls at day 21 in both females (** *p* < 0.01) and males (** *p* < 0.01). This enrichment persisted through day 28, remaining significantly elevated in female TAM-treated rats (** *p* < 0.01) and male TAM-treated rats (* *p* < 0.05), indicating that TAM-associated *Bifidobacterium* expansion was sustained throughout the recovery period. *Lactobacillus* abundance decreased markedly at day 7 in all injured animals before partially recovering during the subacute and chronic phases, with no sustained differences attributable to TAM treatment (Figure 6d). *Romboutsia* abundance declined sharply during the acute post-injury phase and gradually increased thereafter in both sexes (Figure 6e). Although TAM-treated animals generally exhibited higher relative abundance than PLB controls during the recovery period, these differences were variable and not consistently observed across all timepoints. Similarly, *Ruminococcus* abundance decreased in all surgically operated animals at day 7, whereas NAÏVE animals maintained relatively stable populations throughout the study (Figure 6f). Following this initial decline, *Ruminococcus* abundance progressively increased from day 14 through day 28 in both TAM and PLB animals, suggesting recovery of this genus during the subacute and chronic phases after SCI rather than a treatment-specific effect. Collectively, these findings identify *Bifidobacterium* as a TAM-responsive genus, characterized by a delayed expansion that emerges during the subacute phase after SCI and persists through the chronic recovery period in both sexes.

### 2.7. Association Between Gut Bacterial Abundance and Locomotor Recovery Following SCI

**Figure 7:**
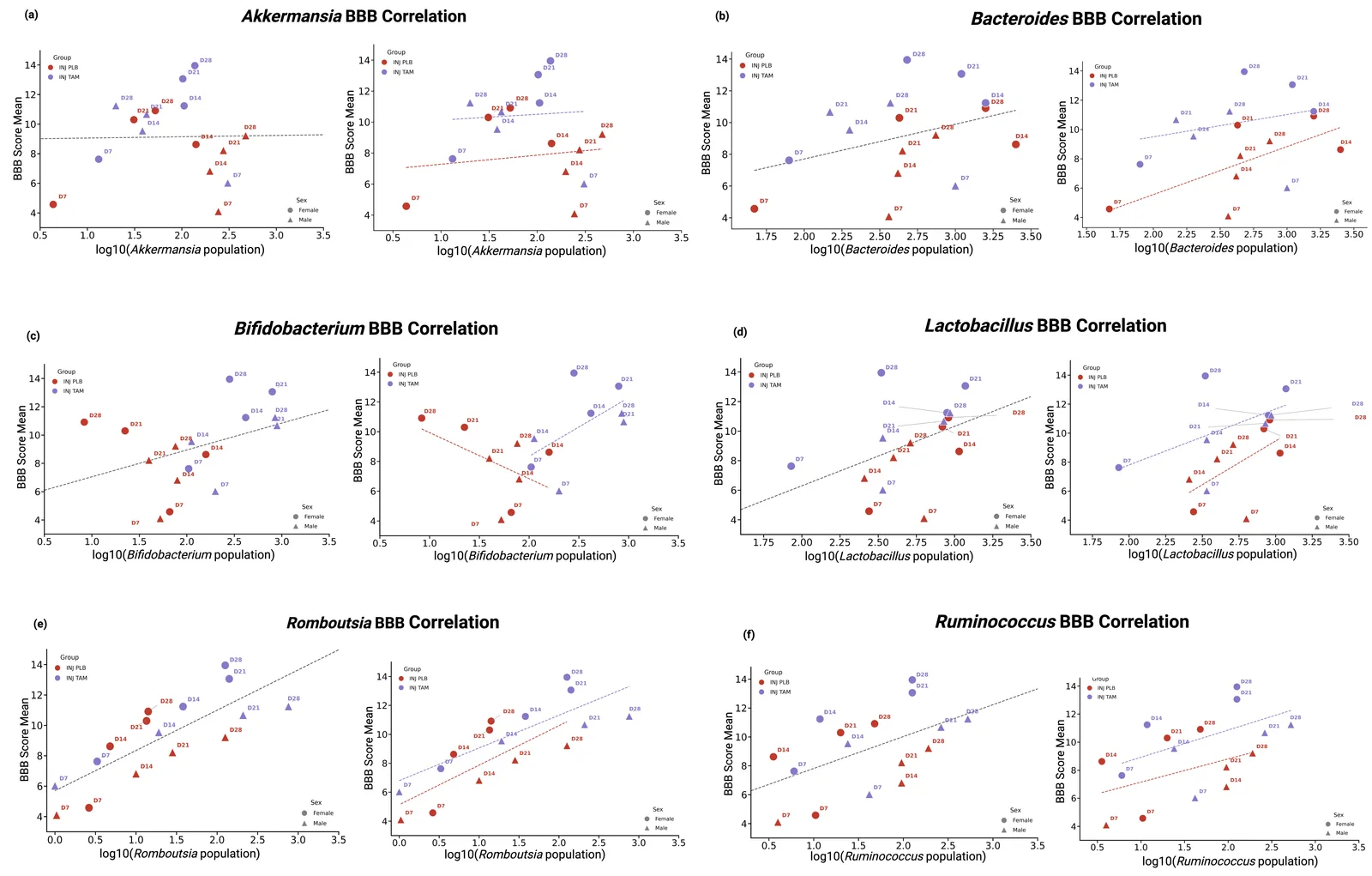
Associations between gut bacterial abundance and locomotor recovery following spinal cord injury. Scatter plots show the relationships between log10-transformed bacterial abundance and mean Basso, Beattie, and Bresnahan (BBB) locomotor scores at 7, 14, 21, and 28 days post-injury (DPI) in female and male INJ PLB and INJ TAM animals for (**a**) *Akkermansia*, (**b**) *Bacteroides*, (**c**) *Bifidobacterium*, (**d**) *Lactobacillus*, (**e**) *Romboutsia*, and (**f**) *Ruminococcus*. Left panels show the overall association across INJ PLB and INJ TAM groups, whereas right panels show group-specific associations. Red symbols represent INJ PLB animals and blue symbols represent INJ TAM animals; circles represent females and triangles represent males. Dashed lines indicate the corresponding correlation trends. Associations between bacterial abundance and BBB scores were evaluated using Spearman rank correlation analysis. Differences between the INJ PLB and INJ TAM Spearman correlation coefficients were assessed using Fisher’s z-test.

To determine whether the abundance of bacterial genera altered following SCI and TAM treatment was associated with locomotor recovery, we conducted an exploratory analysis in which log10-transformed bacterial abundances from group-mean abundances were compared with group-level mean BBB locomotor scores at 7, 14, 21 and 28 DPI, reported for the corresponding sex and treatment groups in earlier studies from our laboratory [26,27]. Because behavioral and microbiome measurements derive from different animals and each group contributes a single BBB value per timepoint, the resulting coefficients describe group-level co-variation and cannot support inference about individual animals (Figure 7). Correlations were first evaluated across the combined INJ PLB and INJ TAM groups and subsequently within each treatment group. Fisher’s z-tests were used to determine whether the strength of the correlations differed between INJ PLB and INJ TAM animals.

*Akkermansia* abundance (Figure 7a) was not associated with BBB scores in the combined analysis (rho = −0.22, *p* = 0.412), and no significant associations were observed within either INJ PLB (rho = −0.12, *p* = 0.779) or INJ TAM animals (rho = −0.14, *p* = 0.736). The correlation coefficients did not differ between treatment groups (Fisher’s z-test, *p* = 0.694). *Bacteroides* (Figure 7b) showed a positive but nonsignificant association with BBB scores in the combined analysis (rho = 0.38, *p* = 0.147). When analyzed by treatment, *Bacteroides* abundance was positively associated with BBB scores in INJ PLB animals (rho = 0.74, *p* = 0.037), whereas the association was not significant in INJ TAM animals (rho = 0.38, *p* = 0.352). However, the difference between these correlations was not significant (Fisher’s z-test, *p* = 0.416). For *Bifidobacterium* (Figure 7c), the combined analysis showed a positive association that approached statistical significance (rho = 0.49, *p* = 0.052). Treatment analysis revealed a significant positive association between *Bifidobacterium* abundance and BBB scores in INJ TAM animals (rho = 0.57, *p* = 0.0139), whereas the association in INJ PLB animals was negative and nonsignificant (rho = −0.50, *p* = 0.320). Despite the opposing direction of these correlations, Fisher’s z-test did not detect a significant difference between treatment groups (*p* = 0.108). *Lactobacillus* (Figure 7d) abundance was significantly positively associated with BBB scores in the combined analysis (rho = 0.55, *p* = 0.026). Positive associations were also observed within INJ PLB (rho = 0.64, *p* = 0.086) and INJ TAM animals (rho = 0.45, *p* = 0.260), although neither reached statistical significance when analyzed separately, and their correlation coefficients did not differ (Fisher’s z-test, *p* = 0.681). The strongest overall association with locomotor recovery was observed for *Romboutsia* (Figure 7e), which showed a robust positive correlation with BBB scores (rho = 0.82, *p* = 0.0001). Positive associations were maintained after analysis by treatment, approaching significance in INJ PLB animals (rho = 0.69, *p* = 0.059) and reaching significance in INJ TAM animals (rho = 0.71, *p* = 0.047). The magnitude of these associations was nearly identical between treatment groups (Fisher’s z-test, *p* = 0.944). Similarly, *Ruminococcus* (Figure 7f) abundance was positively associated with BBB scores in the combined analysis (rho = 0.56, *p* = 0.024), although treatment-specific correlations did not reach significance in either INJ PLB (rho = 0.31, *p* = 0.456) or INJ TAM animals (rho = 0.52, *p* = 0.183), with no difference between groups (Fisher’s z-test, *p* = 0.696). Collectively, these analyses identified positive associations between locomotor recovery and the abundance of several bacterial genera, particularly *Romboutsia*, *Lactobacillus*, and *Ruminococcus*. Treatment analyses further identified significant associations for *Bacteroides* in INJ PLB animals and for *Bifidobacterium* and *Romboutsia* in INJ TAM animals. Notably, *Bifidobacterium* was the only genus exhibiting opposing treatment-specific associations with locomotor recovery, with a negative correlation in INJ PLB animals and a positive correlation in INJ TAM animals, although the difference between these correlations did not reach statistical significance.

### 2.8. Multivariable Linear Modeling Identifies TAM-Associated Increase in Bifidobacterium Following Spinal Cord Injury

**Figure 8:**
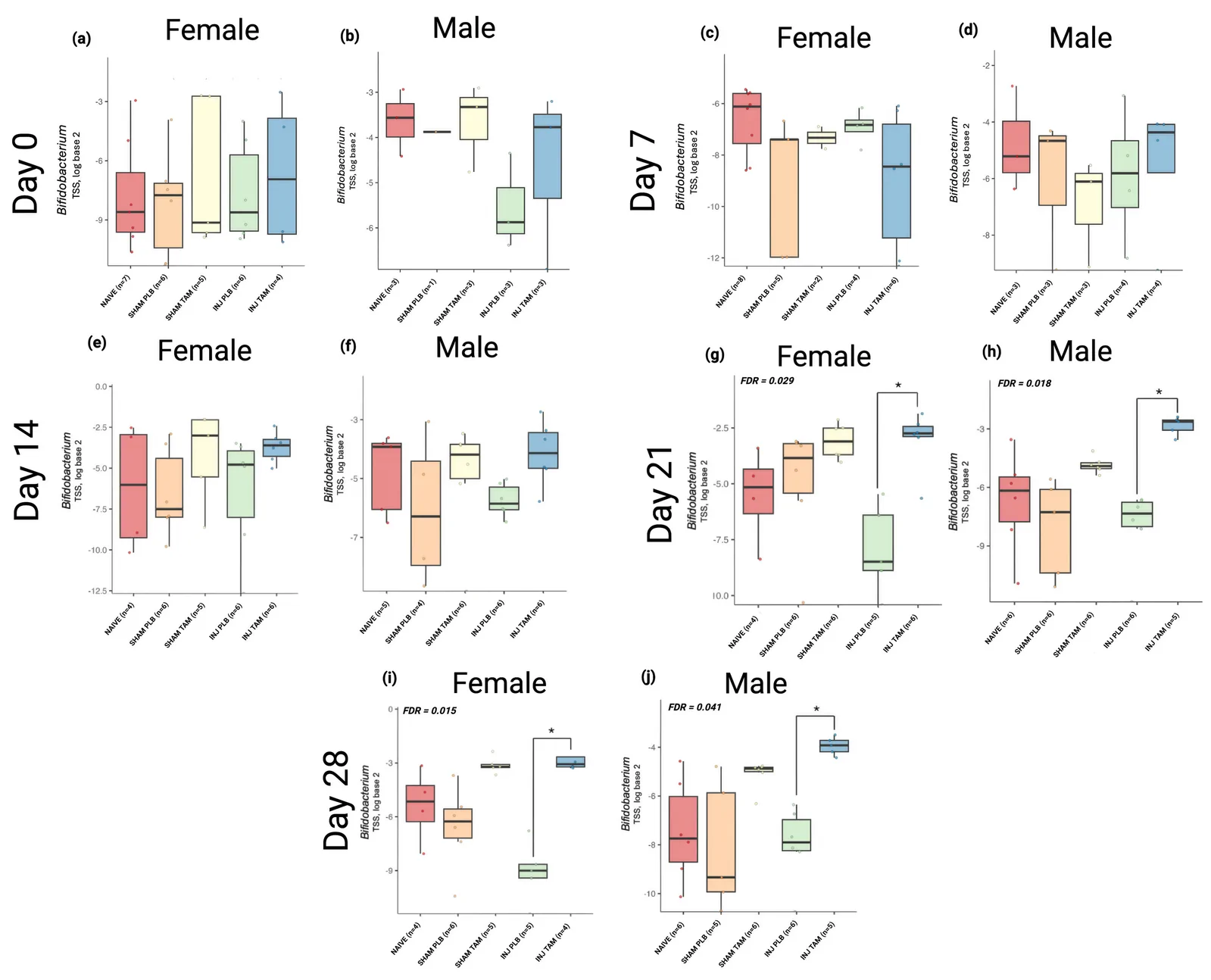
Longitudinal differential abundance of Bifidobacterium following SCI. Relative abundance of *Bifidobacterium* in female (**left**) and male (**right**) rats at baseline (Day 0; **a**,**b**) and post-injury days 7 (**c**,**d**), 14 (**e**,**f**), 21 (**g**,**h**), and 28 (**i**,**j**). Differential abundance was assessed using Multivariable Association with Linear Models (MaAsLin3). Asterisks (*) indicate statistically significant differences following false discovery rate (FDR) correction.

To establish the temporal dynamics of *Bifidobacterium* abundance, longitudinal analyses were performed across all timepoints. No significant differences were detected among groups at baseline (day 0) in either sex (Figure 8a,b), providing no evidence of pre-existing differences in *Bifidobacterium* abundance prior to treatment. At day 7 (Figure 8c,d), *Bifidobacterium* abundance remained uniformly low across groups in both females and males. This reduction is consistent with the combined effects of cefazolin administration and the acute post-SCI phase [40]. At day 14, no significant differences were identified after FDR correction in females or males (Figure 8e,f), though a consistent directional trend of higher *Bifidobacterium* in TAM-treated animals relative to PLB controls was observed in both sexes, suggesting early but not yet statistically consolidated enrichment. By day 21, MaAsLin3 identified a statistically significant TAM-associated increase in *Bifidobacterium* in injured animals of both sexes—females (Figure 8g, FDR = 0.029) and males (Figure 8h, FDR = 0.018)—with INJ TAM exceeding INJ PLB in both cohorts. SHAM TAM groups did not reach significance at this timepoint, a pattern consistent with, but not formally establishing, an injury-dependent treatment effect. This enrichment persisted through day 28, with INJ TAM animals maintaining significantly higher *Bifidobacterium* abundance relative to INJ PLB in females (Figure 8i, FDR = 0.015) and males (Figure 8j, FDR = 0.041). Collectively, these findings confirm a sustained, sex-independent, injury-dependent *Bifidobacterium* enrichment associated with TAM treatment that becomes statistically detectable by day 21 and persists through the chronic recovery period.

### 2.9. Tamoxifen Is Associated with Increased Predicted Glycogen Degradation Pathways Following SCI

**Figure 9:**
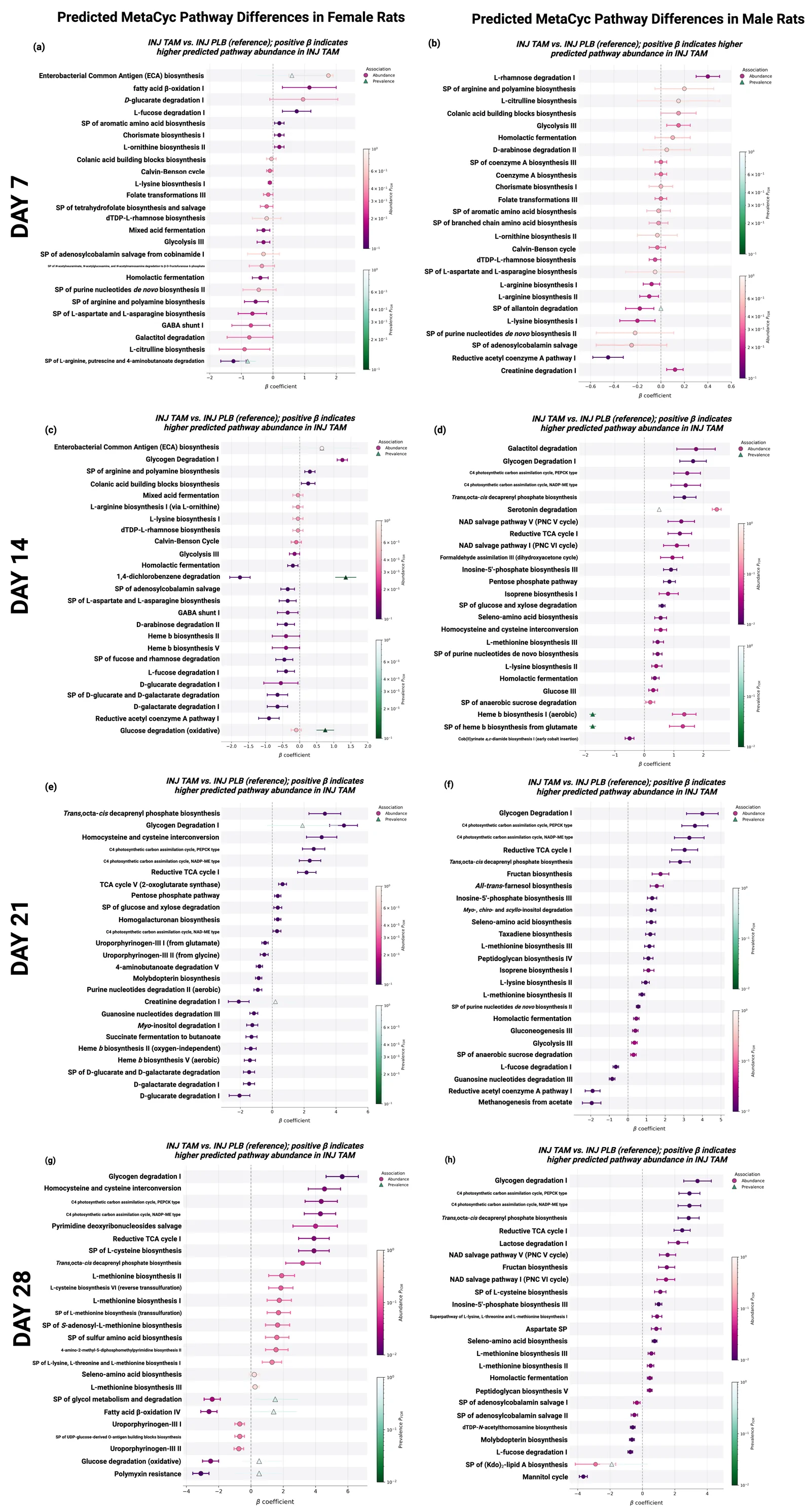
Tamoxifen-associated differences in predicted microbial MetaCyc pathways following SCI. Differential abundance analysis of PICRUSt2-predicted MetaCyc pathways was performed between injured tamoxifen-treated (INJ TAM) and injured placebo-treated (INJ PLB) rats at 7, 14, 21, and 28 days post-injury (DPI), separately for females (**left panels**) and males (**right panels**). INJ PLB was used as the reference group; positive β coefficients indicate greater predicted pathway abundance in INJ TAM animals and negative β coefficients indicate lower predicted abundance relative to INJ PLB. Circles represent pathway abundance associations and triangles represent prevalence associations. Points indicate model β coefficients and horizontal error bars represent 95% confidence intervals. Color intensity represents the corresponding false-discovery-rate (FDR)-adjusted *p*-value. Only pathways meeting the criteria used for inclusion in the differential abundance analysis are displayed.

PICRUSt2 qualitative analysis was performed to investigate whether the taxonomic changes associated with TAM treatment were accompanied by alterations in the predicted functional potential of the gut microbiota following SCI (Figure 9). Differential abundance analysis of predicted MetaCyc pathways revealed time-dependent differences between INJ TAM and INJ PLB animals in both sexes. At 7 DPI, multiple predicted pathways differed between treatment groups, although the overall pattern was heterogeneous, with both positive and negative associations with TAM treatment. This early functional profile was consistent with a period of substantial microbiome perturbation following SCI and did not indicate a uniform directional shift in predicted microbial function. Beginning at 14 DPI, a more distinct TAM-associated functional pattern emerged. Notably, predicted pathways related to glycogen degradation showed increased abundance in INJ TAM animals relative to INJ PLB animals, with this pattern observed in both females and males and maintained at subsequent post-injury timepoints. The recurrence of glycogen-degradation-related pathways from 14 through 28 DPI suggests that TAM-associated microbiome remodeling during the subacute and chronic phases of SCI extends beyond taxonomic composition to alterations in predicted carbohydrate utilization capacity. The emergence of these predicted functional differences at 14 DPI also coincided temporally with the period during which TAM-associated taxonomic changes became increasingly evident in the microbiome. Overall, these findings demonstrate that TAM treatment was associated with dynamic changes in the predicted metabolic potential of the gut microbiota following SCI. Although the specific MetaCyc pathways and magnitude of the associations varied according to sex and post-injury timepoint, the increased representation of glycogen-degradation-related pathways from 14 DPI onward in both sexes represented a recurring functional feature of the TAM-associated microbiome. These results complement the taxonomic analyses by suggesting that the microbial community changes associated with TAM are accompanied by alterations in predicted metabolic functions during recovery following SCI.

## 3. Discussion

The present study demonstrates that tamoxifen (TAM) administration induces a sustained modulation of the gut microbiota following spinal cord injury (SCI), with time-dependent effects that emerge at different stages in female and male rats but result in comparable outcomes. Using a comprehensive longitudinal framework, we show that TAM reshapes global microbial community structure, attenuates post-injury dysbiosis, and selectively promotes the enrichment of specific taxa, with the most pronounced effects observed in injured animals during the subacute and chronic phases following SCI.

Our beta diversity analyses provided the first indication that TAM exerts an influence on overall microbial community composition. This effect was further substantiated by longitudinal beta diversity modeling, which revealed a significant divergence between TAM- and PLB-treated animals over time. Interestingly, the temporal onset of this divergence differed by sex: female rats exhibited a significant microbiome separation as early as 14 days post-injury, whereas males showed a comparable shift at 21 days post-injury (Supplementary Figures S3 and S4, Supplementary Table S1). These results suggest sex-specific changes in microbiome responsiveness to TAM, while ultimately supporting a convergent treatment effect across sexes at 21 and 28 DPI.

Consistent with these community-level changes, F/B ratios in TAM-treated animals were closer to NAÏVE values than those of PLB-treated animals, although the direct TAM-versus-PLB comparison did not reach significance [41]. In our study, a reduction in dysbiosis was particularly evident at 21 days post-injury in both female and male rats (Supplementary Figure S4, Supplementary Table S2), coinciding with the time frame in which TAM-associated beta diversity became most pronounced; also, a highly altered microbiome state was evident at day 7 post-injury. These observations are consistent with previous work from our laboratory showing that the gut microbiota experiences the most significant disruption during the acute post-injury phase, especially at 7 DPI, mainly due to post-operative antibiotic exposure like cefazolin [40]. In that context, the normalization of microbial composition observed in TAM-treated animals at later timepoints suggests a potential role for TAM in facilitating microbiome recovery following early SCI and antibiotic-induced dysbiosis. Further supporting the concept that SCI induces a dysbiotic state in the gut microbiota, a 2023 study by Jing et al. using a murine model reported a significant gut microbial dysbiosis characterized by shifts in community composition accompanied by a marked reduction in short-chain fatty acids (SCFAs), such as acetate, propionate and butyrate [42]. The observed depletion of SCFAs following SCI provides functional evidence that microbiome disruption extends beyond taxonomic changes and reflects a loss of beneficial microbial activity. 

Taxonomic profiling further supported the microbiome modulatory effects of TAM. At the phylum level, all TAM-treated groups (SHAM and INJ) exhibited increased relative abundance of Actinobacteria, a phylum associated with intestinal health [43]. At the genus level, the TAM-associated taxonomic changes observed in both sexes are consistent with a shift towards a healthier and anti-inflammatory gut microbiota state. Enrichment of *Bifidobacterium* and *Parabacteroides*—the two genera consistently increased in TAM-treated animals here—has been linked to enhanced intestinal barrier function, increased SCFA production, and attenuation of pro-inflammatory signaling in the gut [44,45,46,47]. *Lactobacillus*, by contrast, declined in all injured animals in this study (Section 2.6). Together, these findings are consistent with the interpretation that TAM treatment promotes a gut microbial environment associated with metabolic homeostasis and immune regulation. 

Cross-sectional analysis revealed differential abundance across multiple genera following SCI and TAM administration. Although population changes in *Roseburia* and *Desulfovibrio* were observed in TAM-treated female rats, along with *Blautia* and *Streptococcus* in TAM-treated male rats, these differences were restricted to individual post-injury timepoints and were not maintained longitudinally or consistently across both sexes. Therefore, our discussion focused primarily on bacterial genera exhibiting sustained or biologically relevant temporal changes throughout the recovery period. *Akkermansia* exhibited a transient post-injury increase in male treatment groups that was subsequently modulated by TAM in a sex-specific manner. In contrast, *Lactobacillus* abundance declined markedly at day 7 in injured animals, with the most pronounced reduction observed in TAM-treated females. This finding is consistent with our previous study, in which we demonstrated a marked reduction in *Lactobacillus* abundance during the first week after SCI following cefazolin administration [40]. Together, these observations suggest that the early depletion of *Lactobacillus* primarily reflects acute SCI-associated dysbiosis combined with post-operative antibiotic exposure rather than a direct effect of TAM. Notably, the comparatively high overall abundance of *Lactobacillus* observed in females is consistent with previous reports of greater fecal *Lactobacillus* abundance in female compared with male rats [48,49,50]. However, because taxonomic assignments in the present study were generated using SILVA v138.1, the *Lactobacillus* designation reflects the taxonomy implemented in this reference database and should be interpreted in the context of the extensive taxonomic reorganization of the genus reported in 2020 [51]. *Bacteroides* fluctuated after injury but showed no consistent TAM-associated difference at any timepoint, suggesting that its dynamics reflect the injury and post-operative course rather than treatment. A similar temporal pattern was observed for *Romboutsia*, with abundance markedly reduced during the acute post-injury period in both sexes, followed by a gradual recovery at later timepoints. Although *Romboutsia* abundance tended to be higher in TAM-treated animals, particularly in females at 14 and 21 DPI and in males at 28 DPI, these differences were not consistently maintained throughout the study. Likewise, *Ruminococcus* abundance decreased sharply after SCI in all experimental groups by day 7, whereas NAÏVE animals of both sexes maintained relatively stable populations throughout the study. Following this acute decline, *Ruminococcus* gradually increased from day 14 through day 28 in both TAM- and PLB-treated animals, suggesting recovery of this genus during the subacute and chronic phases after SCI rather than a treatment-specific effect. Among all genera examined, *Bifidobacterium* emerged as the most statistically robust and temporally consistent TAM-associated responder. 

The increase in *Bifidobacterium* abundance was observed in both sexes, although the response emerged earlier in females and reached comparable levels in males by day 21. Importantly, Microbiome Multivariable Associations with Linear Models (MaAsLin3) identified statistically significant differences in *Bifidobacterium* abundance when directly comparing injured TAM-treated rats (INJ TAM) with PLB-treated controls (INJ PLB). These differences were most pronounced at 21 and 28 days after SCI in both sexes, underscoring the relevance of injury in shaping TAM-associated microbiome effects. TAM increased *Bifidobacterium* abundance in both SHAM and INJ animals, and the effect appeared larger in INJ animals, raising the possibility that TAM preferentially promotes beneficial microbial expansion in an injury-compromised gut.Importantly, TAM-associated microbiome changes were not restricted to injured animals, as *Bifidobacterium* enrichment was also observed in SHAM TAM animals, indicating that TAM can modulate gut microbial composition independently of SCI. However, the magnitude of this response was greater in the post-injury setting, with INJ TAM animals exhibiting the most pronounced enrichment at later timepoints and significantly greater *Bifidobacterium* abundance than INJ PLB animals at 21 and 28 DPI in both sexes. Thus, our findings support a broader microbiome-modulatory effect of TAM while suggesting that the altered post-SCI intestinal environment may influence the magnitude or trajectory of this response. 

Our focus on *Bifidobacterium* is further supported by its well-established roles in anti-inflammatory processes through the production of short-chain fatty acids and maintenance of mucosal integrity, both of which are critically disrupted following SCI. The potential relevance of *Bifidobacterium* to CNS function is further supported by studies using germ-free animal models. In a 2020 study, Luck et al. demonstrated that neonatal colonization of germ-free mice with a defined consortium of four *Bifidobacterium* species influenced microglial function, synaptic organization, and neuronal activity. Notably, germ-free mice exhibited increased synaptic density and reduced Purkinje cell firing rates compared with *Bifidobacterium*-colonized mice, whereas colonization with *Bifidobacterium* was associated with normalization of synaptic density and neuronal activity. These findings provide experimental evidence that members of the genus *Bifidobacterium* can influence neural circuit development and function through microbiota–CNS interactions [52]. Further, in a 2016 study using rodents, Kigerl et al. showed how the administration of the probiotic VSL#3, rich in *Lactobacillus* and *Bifidobacterium* strains, improved neuroprotection and locomotor recovery after SCI. [53]. Lastly, previous work from our laboratory demonstrated that TAM-treated rats exhibit improved locomotor recovery by 14 days post-SCI [26,27], a timepoint that coincides precisely with the initial emergence of *Bifidobacterium* enrichment observed in the present study. This temporal correspondence indicates that TAM-associated microbiome remodeling and previously reported functional improvement emerge over a similar post-injury period; however, it does not establish a mechanistic relationship between these outcomes.

Although TAM could theoretically exacerbate SCI-associated dysbiosis through interactions with the estrobolome, we observed no evidence of aggravated dysbiosis; estrogen-metabolizing gene content and circulating estrogen metabolites were not measured, so this pathway remains untested and exploratory. Instead, in our SCI model, TAM treatment was associated with attenuation of post-injury dysbiosis and a sustained enrichment of *Bifidobacterium*, the most robust and consistent treatment-associated microbial signature identified in both sexes. These observations suggest that, within the context of moderate SCI, TAM does not induce the pro-inflammatory microbial shift that might be anticipated from perturbation of estrogen-microbiota interactions. Our findings contrast with previous reports describing TAM-associated dysbiosis in other experimental settings. However, those studies were conducted under substantially different biological and experimental conditions, including xenograft mouse models, high-dose TAM administration to induce hepatotoxicity, and fecal microbiota transplantation models using human donor material, all of which profoundly alter the native gut microbial ecosystem [48,49,50]. Furthermore, TAM was primarily administered in those studies as a long-term anticancer therapy rather than as a short-term neuroprotective intervention following CNS injury. In contrast, the present study employed a clinically relevant moderate contusion SCI model characterized by a unique inflammatory and autonomic environment. Collectively, differences in dosing regimen, disease context, injury severity, and baseline microbial composition likely account for the divergent microbiome outcomes reported across studies and underscore the context-dependent effects of TAM on the gut microbiota. 

Additionally, the beneficial effect of TAM on locomotor recovery following SCI is further supported by previous experimental studies. In a 2009 study, Tian et al. demonstrated that TAM administration following spinal cord contusion significantly improved locomotor function in rats compared with vehicle-treated animals, as assessed using the BBB locomotor rating scale. This functional improvement was accompanied by attenuation of inflammation-mediated secondary damage, including reduced microglial activation, neuronal apoptosis, and myelin loss [54]. Consistent with these findings, previous studies from our laboratory by Colón et al. demonstrated improved locomotor recovery following continuous TAM administration in both female and male rats with moderate contusion SCI. In female rats, TAM administered immediately or 24 h after SCI significantly improved BBB locomotor scores compared with PLB-treated animals, whereas continuous TAM treatment initiated immediately or 6 h after SCI similarly improved locomotor recovery in male rats [26,27]. The mean BBB scores reported in these previous studies from our laboratory were used in the present correlation analyses to examine the relationship between bacterial abundance and functional recovery. Notably, among the bacterial genera examined, *Bifidobacterium* was the only genus exhibiting opposing treatment-specific associations with locomotor recovery, with bacterial abundance negatively correlated with mean BBB scores in INJ PLB animals but positively correlated with mean BBB scores in INJ TAM animals. Although the difference between these treatment-specific correlations did not reach statistical significance, the positive association observed in TAM-treated animals is particularly noteworthy given the sustained TAM-associated enrichment of *Bifidobacterium* identified in both sexes in the present study. Together with the established beneficial effect of TAM on locomotor recovery after SCI, these findings identify *Bifidobacterium* as a potentially relevant microbial correlate of TAM-associated functional recovery. However, because these associations were derived from group-level mean BBB scores and the treatment-specific correlations were not significantly different, further studies using paired microbiome and behavioral measurements from individual animals will be necessary to determine whether *Bifidobacterium* contributes directly to neurological recovery.

The predicted functional changes identified by PICRUSt2 provide additional evidence that TAM-associated remodeling of the gut microbiota may extend beyond taxonomic composition to microbial metabolic potential. Notably, glycogen degradation I emerged as a recurrent TAM-associated pathway beginning at 14 DPI and remained increased at later post-injury timepoints in both sexes. This temporal pattern is particularly interesting given the concurrent TAM-associated enrichment of *Bifidobacterium* observed in the present study. Members of the genus *Bifidobacterium* possess extensive carbohydrate utilization capabilities, including pathways involved in the degradation of complex carbohydrates and glycogen [55]. Comparative genomic analyses have identified glycogen degradation I and II pathways across multiple intestinal *Bifidobacterium* species, and transcriptomic studies have demonstrated activation of genes comprising the glycogen degradation I pathway during growth on glycogen, supporting the capacity of these bacteria to utilize glycogen as a carbohydrate source [56,57]. Glycogen degradation generates intermediates that can enter central carbohydrate metabolism; in bifidobacteria, carbohydrate-derived substrates can ultimately be processed through the characteristic fructose-6-phosphate phosphoketolase pathway, or “bifid shunt,” which supports production of fermentation end products including acetate and lactate [58,59]. Therefore, the parallel emergence of increased predicted glycogen degradation potential and *Bifidobacterium* enrichment after 14 DPI may reflect a TAM-associated shift toward greater microbial carbohydrate utilization capacity during SCI recovery. However, because PICRUSt2 infers functional potential from 16S rRNA gene profiles rather than directly measuring gene expression or metabolic activity, the present analysis cannot establish that *Bifidobacterium* was directly responsible for the observed increase in glycogen degradation pathways. Moreover, because the present 16S rRNA gene sequencing approach primarily resolves these changes at the genus level, the specific *Bifidobacterium* species underlying this enrichment and their functional activity remain unknown; higher-resolution metagenomic and metabolomic approaches will be required to determine the biological significance of this taxonomic shift.

This temporal alignment between previously reported functional outcomes and the microbiome changes observed in the present study raises the possibility that TAM-associated modulation of beneficial microbial taxa accompanies biological processes, which could be compatible with locomotor recovery after SCI. Since microbiome composition and locomotor outcomes were measured in different animals, bacterial abundances from the present cohort were compared with group-level mean BBB scores obtained from previously published cohorts from our laboratory. Therefore, these analyses demonstrate only group-level temporal co-variation and cannot establish an individual-level association or a causal contribution of the gut microbiota, including *Bifidobacterium*, to TAM-mediated locomotor recovery. The observed correspondence should therefore be considered hypothesis-generating, and studies incorporating concurrent microbiome and behavioral measurements, together with targeted microbiome manipulation, will be required to determine whether TAM-associated microbial changes contribute mechanistically to neurological recovery after SCI.

Lastly, post-operative cefazolin exposure should also be considered when interpreting these longitudinal microbial changes. In our previous study using the same 7-day cefazolin regimen in an experimental SCI model, the most pronounced microbiome disruption occurred during the acute post-injury period, particularly at 7 DPI, followed by a tendency toward greater microbial stability at later timepoints after antibiotic treatment had ended [40]. A similar temporal pattern was observed here, as *Bifidobacterium* abundance remained low across groups at 7 DPI and TAM-associated enrichment emerged beginning at 14 DPI, seven days after cefazolin treatment ended. Because all surgical TAM- and PLB-treated animals received the same cefazolin regimen, antibiotic exposure alone is unlikely to account for the subsequent treatment-associated divergence; however, the data also support the possibility that TAM facilitates or accelerates microbiome recovery following antibiotic-associated disturbance.

## 4. Materials and Methods

### 4.1. Animal Housing

Adult female (~238.29 g) and male Sprague–Dawley rats (~313.48 g) were obtained from Hilltop Lab Animals (Scottdale, PA, USA). Upon arrival, animals were pair-housed during the acclimation phase and individually housed throughout experimental procedures and post-operative recovery. Rats were maintained under standard environmental conditions (12:12 h light/dark cycle) with unrestricted access to chow (Harlan Teklad, Madison, WI, USA) and water. All procedures were conducted in accordance with protocols approved by the Institutional Animal Care and Use Committee (IACUC) of the University of Puerto Rico, Medical Science Campus.

### 4.2. Spinal Cord Injury Model and Tamoxifen Treatment

All surgical procedures were performed under sterile conditions. A total of 61 animals were included in this study (32 female and 29 male): 24 female and 23 male rats underwent surgical procedures, and 8 female and 6 male rats served as naïve controls without surgical or pharmacological intervention. Successful induction of moderate spinal cord injury (SCI) was confirmed by open-field locomotor assessment using the Basso, Beattie, and Bresnahan (BBB) locomotor rating scale. At 48 h post-injury, animals in the SCI groups exhibited severe hindlimb locomotor deficits, with BBB scores ≤ 2, consistent with the expected phenotype following moderate contusive SCI [60]. All surgical procedures were conducted following previously established and published protocols [61,62]. Briefly, anesthesia was induced with a cocktail of ketamine (36 mg/kg), xylazine (3.6 mg/kg), and acepromazine (1.6 mg/kg). Animals were assigned to groups by random numbering stratified by sex. Surgeons were blinded to treatment allocation; personnel performing DNA extraction, sequencing and bioinformatic analysis were blinded to group identity until analysis was complete. All SHAM animals received only a laminectomy at T10 without contusion, followed by closure of the incision. In the INJ groups, the laminectomy was followed by a moderate contusion using the NYU/MASCIS impactor device in which a 10 g weight was dropped from a height of 12.5 mm on the exposed spinal cord and compression was maintained for 5 s. TAM was administered using the sustained release of a 0.71 mg/day subcutaneous pellet designed to provide continuous drug delivery for 21 days. Animals were followed until 28 days post-injury to evaluate longitudinal microbiome changes during treatment and during the week following completion of pellet drug release, when TAM and its active metabolites remain biologically active [63]. Immediately after the injury, animals received a subcutaneous implantation of either a 15 mg TAM pellet or placebo (PLB) pellet (Innovative Research of America Sarasota, FL, cat# E-361) in the mid-scapular region. Post-surgery, all animals received subcutaneous saline, ophthalmic lubricant, and cefazolin (25 mg/kg, intraperitoneally, twice daily for 7 days) for infection prophylaxis. Buprenorphine (0.05 mg/kg, intraperitoneally) was administered twice daily for 3 days for analgesia. Manual bladder expression was provided to injured animals until spontaneous voiding resumed. Enrichment with paper-based materials and cereal treats was included during the study.

### 4.3. Fecal Sample Collection, DNA Extraction and 16S rRNA Sequencing

Fecal samples (~250 mg per animal) were collected aseptically at five timepoints: baseline (day 0) and days 7, 14, 21, and 28 post-intervention. To prevent cross-contamination, sterilized tweezers were rinsed with alcohol between each collection. All samples were immediately stored at −80 °C until further processing.

Genomic DNA (gDNA) was extracted using the DNeasy PowerSoil Kit (QIAGEN, Germantown, MD, USA) according to the manufacturer’s instructions. Briefly, 800 µL of CD1 lysis buffer was added to each PowerBead Pro Tube® containing the fecal sample. For homogenization, tubes were vortexed horizontally at maximum speed for 10 min. Subsequently, 200 µL of CD2 was added to precipitate the contaminant, followed by 60 µL of CD3 to facilitate DNA binding to the silica membrane. Wash steps included 500 µL of EA buffer and 500 µL of ethanol-based CD5 solution. DNA was eluted with 50 µL of CD6, yielding approximately 30 µL of purified gDNA per sample. DNA concentration was measured using the Qubit™ 1X dsDNA High Sensitivity Assay Kit on a Qubit Flex Fluorometer (Thermo Fisher Scientific, Waltham, MA, USA). The V4 region of the 16S rRNA gene was amplified using primers 515F (5′-GTGCCAGCMGCCGCGGTAA-3′) and 806R (5′- GGACTACHVGGGTWTCTAAT- 3′). Amplicons were sequenced on the Illumina MiSeq (Illumina, San Diego, CA, USA) using 2 x 250 bp paired-end sequencing (Argonne National Laboratory, IL, USA). Quality control of 16S rRNA demultiplexed amplicons was performed in QIITA (v2025.07) [64] and downstream analyses were conducted using QIIME 2 (v.2023.7) [65], R (v4.3.1), and MicrobiomeAnalyst 2.0 [66].

The experimental design initially included six animals per sex per experimental group (Figure 1a). Fecal samples were collected longitudinally from the same animals at 0, 7, 14, 21, and 28 DPI; therefore, sample sizes reported at individual timepoints represent fecal samples rather than unique animals. The number of samples included in individual downstream microbiome analyses varied across groups and timepoints following sequencing quality control and data preprocessing. Specifically, microbiome analyses were performed after rarefaction to a sequencing depth of 4000 reads per sample (Supplementary Figure S5), and samples that did not meet the minimum sequencing depth or other quality-control requirements were excluded from the corresponding analyses. Therefore, sample sizes reported in individual figures represent the number of samples retained for that specific analysis and may differ from the initial number of animals enrolled in each experimental group. The number of samples included in each analysis is indicated in the corresponding figure legends.

### 4.4. Microbiome Population Analysis

#### 4.4.1. Beta Diversity

Taxonomic classification was performed using the QIIME 2 feature-classifier plugin (classify-sklearn) with the SILVA reference database (v138.1). Beta Diversity ordination plots were generated using the MicrobiomeAnalyst web platform [66]. To visualize differences in microbial community structure, aPrincipal Coordinates Analysis (PCoA) was employed. Group-level differences were evaluated by PERMANOVA (999 permutations) on Bray–Curtis dissimilarities, with permutations constrained within animals to respect the repeated-measures design. Homogeneity of multivariate dispersion was tested with PERMDISP, and pairwise PERMANOVA *p*-values were corrected with the Benjamini–Hochberg procedure.

#### 4.4.2. Firmicutes/Bacteroidetes Ratio

The Firmicutes–Bacteroidetes Ratio (F/B) has been widely proposed as an indicator of gut microbiota dysbiosis. To examine alterations in this metric, F/B ratios were visualized using boxplots generated with the ggplot2 [67] package in R, with data handling performed using tidyverse [68]. Statistical differences among groups were evaluated using the non-parametric Kruskal–Wallis test, followed by Dunn’s post hoc multiple comparisons test.

#### 4.4.3. Taxonomic Profiles and Longitudinal Analysis

Taxonomic profiles and microbial biomarker analyses were conducted to identify bacterial genera associated with the beneficial effects of TAM on both sexes (Supplementary Figures S1 and S2). Differential microbial taxa among experimental groups were identified using the Linear Discriminant Analysis Effect Size (LEfSe) approach implemented in MicrobiomeAnalyst [66]. Taxa with an LDA score ≥ 1.0 and a *p*-value < 0.05 were considered significant. Heatmaps were generated to visualize taxa abundance across all five timepoints using Euclidean distance and Ward’s linkage algorithm. To assess longitudinal changes in the gut microbial community following SCI and TAM administration, a first-distances volatility analysis was performed using the q2-longitudinal plugin in QIIME 2 [69]. The q2-longitudinal plugin provides a specialized suite of statistical and visualization tools—including linear mixed effects modeling, volatility tracking, and maturity index prediction—specifically designed to analyze how microbial communities evolve across time series and longitudinal study designs. In our study, to quantify longitudinal microbial volatility, we calculated the dissimilarity of each post-injury sample, distances were calculated between consecutive sampling timepoints for each animal (day 7 vs. day 0, day 14 vs. day 7, day 21 vs. day 14, and day 28 vs. day 21). These distance values were visualized across days 7, 14, 21, and 28 using volatility plots, allowing a comparison of temporal shifts across sex and treatment cohorts (NAÏVE, SHAM PLB, SHAM TAM, INJ PLB, and INJ TAM). Longitudinal abundance trajectories were further evaluated for representative bacterial genera that exhibited sufficient prevalence and consistent relative abundance across sampling timepoints to permit meaningful longitudinal analysis. These included *Akkermansia, Bacteroides, Bifidobacterium, Lactobacillus, Romboutsia,* and *Ruminococcus*. Mean relative abundance was plotted at each sampling time, with shaded regions representing the standard deviation (SD). Statistical comparisons between TAM- and placebo-treated animals were performed separately for each sex at individual timepoints using two-sided Mann–Whitney U tests on individual animal relative abundance values. Statistical significance was defined as *p* < 0.05. Lastly, to validate the observed temporal trends in microbial genera significantly associated with treatment and sex, a multivariable association analysis was performed using MaAsLin3 [70] as follows: feature ~ treatment + injury + sex + timepoint + treatment:injury + (1 | animal_ID), with INJ PLB as the reference group. Abundances were total-sum-scaled (TSS) and log-transformed; features were retained if present in ≥30% of samples at a relative abundance ≥ 0.1%. Both abundance and prevalence models were fitted, and associations with Benjamini–Hochberg q < 0.05 were considered significant.

### 4.5. Correlation Analysis Between Bacterial Abundance and Locomotor Recovery

To evaluate associations between gut bacterial abundance and locomotor recovery following spinal cord injury (SCI), Spearman rank correlation analyses were performed between log10-transformed bacterial abundance and Basso, Beattie, and Bresnahan (BBB) locomotor scores. BBB scores were obtained from a previous behavioral experiment conducted in our laboratory using corresponding male and female SCI treatment groups [26,27]. Mean BBB scores at 7, 14, 21, and 28 days post-injury (DPI) were matched to bacterial abundance values from the corresponding sex, treatment group, and timepoint. Correlation analyses were restricted to injured animals receiving placebo (INJ PLB) or tamoxifen (INJ TAM). Statistical analyses were performed in R. For each bacterial genus, Spearman correlations were assessed across the combined INJ PLB and INJ TAM groups and separately within each treatment group, with both sexes included in the analyses. Spearman rank correlation coefficients (rho) and corresponding two-sided *p*-values were calculated using the cor.test() function with method = spearman. To determine whether the strength of the bacterial abundance–BBB associations differed between treatment groups, the treatment-specific Spearman correlation coefficients were compared using Fisher’s r-to-z transformation. Comparisons were performed in R using the cocor package and the cocor.indep.groups() function with the Fisher (1925) test and a two-sided alternative hypothesis [71]. The Spearman rho values and corresponding numbers of observations for the INJ PLB and INJ TAM groups were used for these comparisons. A *p*-value < 0.05 was considered statistically significant. Scatter plots were generated using the ggplot2 and ggrepel R packages [67]. Log10-transformed bacterial abundance was plotted against mean BBB score, with INJ PLB and INJ TAM groups distinguished by color and sex distinguished by point shape. Individual observations were labeled according to DPI.

### 4.6. Prediction and Differential Analysis of Microbial Functional Pathways

The functional potential of the gut microbiota was inferred from 16S rRNA gene sequencing data using Phylogenetic Investigation of Communities by Reconstruction of Unobserved States 2 (PICRUSt2) [72]. Representative amplicon sequence variant (ASV) sequences and their corresponding abundance tables were used as input for the PICRUSt2 pipeline. ASV sequences were phylogenetically placed into the PICRUSt2 reference tree, followed by prediction of gene family abundances and reconstruction of microbial metabolic pathways. Predicted pathway abundances were generated using the MetaCyc pathway database, and unstratified pathway abundance tables were used for downstream statistical analysis. To identify TAM-associated differences in predicted microbial functional potential following SCI, differential abundance analyses were performed separately for female and male rats at 7, 14, 21, and 28 DPI. At each timepoint, analyses were restricted to injured animals receiving placebo (INJ PLB) or TAM (INJ TAM). Predicted MetaCyc pathway abundances were analyzed in R using Microbiome Multivariable Associations with Linear Models 3 (MaAsLin3) [70]. Pathway abundance data were normalized using total sum scaling (TSS) and log-transformed prior to modeling. INJ PLB served as the reference group for comparison with INJ TAM. Accordingly, positive β coefficients indicate greater predicted pathway abundance in INJ TAM relative to INJ PLB, whereas negative β coefficients indicate greater predicted pathway abundance in INJ PLB relative to INJ TAM. Multiple-testing correction was performed using the false discovery rate (FDR), and associations with FDR-adjusted *p*-values (q-values) < 0.05 were considered statistically significant.

## 5. Conclusions

This study demonstrates that tamoxifen (TAM) administration induces a sustained modulation of the gut microbiota following spinal cord injury (SCI), characterized by progressive enrichment of beneficial microbial taxa. Using a longitudinal, sex inclusive framework, we show that TAM alters global microbial community structure, attenuates post-SCI dysbiosis, and selectively promotes *Bifidobacterium* enrichment, with effects most pronounced in SCI animals during the subacute and chronic phases of recovery. Although microbiome responses emerged earlier in females than in males, the persistence of TAM-associated effects converges in both sexes. Multivariable modeling identified *Bifidobacterium* as the most consistent and statistically significant genus associated with TAM treatment, particularly in SCI animals at 21 and 28 days after injury. These findings align with prior work demonstrating severe microbiome disruption during the acute phase after SCI, partly driven by post-operative antibiotic exposure, and suggest that TAM may facilitate microbiome recovery following early dysbiosis. Importantly, integration of previously published mean Basso, Beattie, and Bresnahan (BBB) locomotor scores with bacterial abundance revealed opposing treatment-specific associations for *Bifidobacterium*, with a negative correlation in INJ PLB animals and a positive correlation in INJ TAM animals. Although the difference between these correlations did not reach statistical significance, this pattern identifies *Bifidobacterium* as a microbial taxon of particular interest in relation to TAM-associated functional recovery. PICRUSt2 analysis further indicated that TAM-associated microbiome changes extended to predicted microbial function. Glycogen-degradation-related pathways increased in INJ TAM animals beginning at 14 DPI and persisted in both sexes, coinciding with *Bifidobacterium* enrichment and suggesting altered microbial carbohydrate metabolism. However, as PICRUSt2 infers functional potential from 16S rRNA profiles, these findings require validation through direct functional analyses. 

A limitation of this study was the reduced sample size in male cohorts at day 7 post-SCI, mainly due to stringent quality control criteria applied during genomic data processing. This limitation was mitigated by the consistency of microbiome patterns observed at later timepoints, where adequate sample sizes were available and TAM-associated effects were reproducibly detected across multiple analytical frameworks, indicating that reduced power at day 7 in males did not influence the overall conclusions. It is also important to acknowledge that individual food intake was not quantitatively assessed throughout the experimental period. Although all animals had ad libitum access to the same standard laboratory chow and water, SCI-associated changes in appetite or food consumption could potentially contribute to alterations in gut microbiota composition. Additionally, body weight was recorded at the beginning of the experiment but was not longitudinally monitored at each experimental timepoint, limiting our ability to determine whether changes in body weight were associated with the observed microbiome alterations. Therefore, potential differences in dietary intake and body weight cannot be completely excluded as factors influencing the observed microbial changes. Lastly, a limitation is that the present study focused on taxonomic changes in the gut microbiota and did not directly assess the functional consequences of these alterations. Although TAM treatment was associated with sustained changes in microbial community composition and enrichment of *Bifidobacterium*, the effects of these changes on microbial metabolism and host physiology remain to be established. Several further limitations apply. First, the locomotor scores used in the correlation analyses are group means from previously published cohorts rather than measurements from the animals sequenced here, so those associations are exploratory. Second, several cross-sectional comparisons pooled repeated samples from the same animals. Third, all surgical animals received cefazolin, which confounds treatment effects at early timepoints. Fourth, animals were individually housed after a pair-housed acclimation, and cage effects on microbiota composition cannot be excluded. Fifth, TAM and metabolite exposure was not verified analytically through metabolomics. 

Future integration of microbiome profiles with SCFAs and other metabolite analyses, colon histology and markers of intestinal barrier integrity, and systemic inflammatory factors will be necessary to determine the functional significance of TAM-associated microbiome remodeling following SCI. Future experiments are planned to analyze the effect of TAM on intestinal epithelial integrity after SCI, whether there is any change in the level of SCFAs from fecal samples in our groups, and whether there are significant changes in the pro- or anti-inflammatory cytokines in the presence of TAM after SCI, which could help to elucidate the neuroprotective effects of this drug.

Together, these findings demonstrate that TAM treatment following SCI is associated with coordinated taxonomic and predicted functional remodeling of the gut microbiota. The sustained enrichment of *Bifidobacterium*, its positive association with locomotor recovery in TAM-treated animals, and the concurrent emergence of predicted glycogen degradation pathways identify a potential microbial–metabolic signature associated with TAM treatment. These findings position the gut microbiota, and *Bifidobacterium* in particular, as a potential component of the biological response to TAM following SCI and provide a foundation for mechanistic studies investigating whether microbiome modulation contributes to TAM-associated neuroprotection and functional recovery.

## Data Availability

The 16S rRNA gene sequences can be found in the QIITA study ID 16051. They are also available in the European Nucleotide Archive ENA Project (ERP196105).

## Supplementary Materials

The following supporting information can be downloaded at: https://www.mdpi.com/article/10.3390/ijms27198729/s1.
